# Modelling hospital operations: insight from using data from paper registries in the obstetrics ward at a hospital in Addis Ababa, Ethiopia

**DOI:** 10.1136/bmjgh-2018-001281

**Published:** 2019-05-16

**Authors:** Benjamin Bigelow, Dawit N Desalegn, Joshua A Salomon, Stéphane Verguet

**Affiliations:** 1 Global Health and Population, Harvard University T H Chan School of Public Health, Boston, Massachusetts, USA; 2 Global Public Health and Primary Care, University of Bergen, Bergen, Norway; 3 Center for Medical Ethics and Priority Setting, Addis Ababa University College of Health Sciences, Addis Ababa, Ethiopia; 4 Medicine, Stanford University School of Medicine, Palo Alto, California, USA

**Keywords:** health systems, hospital-based study, maternal health, health services research

## Abstract

In the Ethiopian health system, operations management techniques have been underutilised. Although previous research has outlined limitations of paper-based patient records, few studies have examined their potential utility for improving management of hospital operations. In this paper, we used data collected from paper registries in an Ethiopian obstetrics ward at Addis Ababa’s Tikur Anbessa Specialized Hospital, Ethiopia’s largest university hospital, to model the ward’s operations. First, we attempted to identify predictors of lengthy stays and readmissions among women giving birth: few predictors were deemed significant. Second, time series methods for demand forecasting were applied to the data and evaluated with several error metrics, and these forecasts were improvements over baseline methods. We conclude with recommendations on how the obstetrics ward could incorporate our modelling approaches into their daily operations.

**Summary box**

- Operations management techniques have been underutilised in global health, particularly in low-income and middle-income countries.
- Little research has examined the utility of existing data sources for improving hospital operations.
- Some operational insights can be drawn from administrative data sources, but problems with data quality and missing data limit their usefulness.
- Before operations management techniques can be successfully implemented, improvements in data quality must be prioritised by health facility and hospital administrators.

## Introduction

In recent years, health system strengthening has become a major focus of policymakers and researchers in global health. Guiding this research are several health system frameworks such as the one developed by the WHO. Among the ‘building blocks’ of the health system are service delivery, workforce, leadership and governance; within the framework, these foundational elements should contribute to efficiency, improved health and financial risk protection.^1^ As Bradley *et al* 2 noted, however, a key component—namely management—has been recognised only in the context of service delivery, despite the strong evidence linking management capacity to health system performance in low-income and middle-income countries.

Several studies of hospital management and quality improvement have been undertaken in Ethiopia.^3–6^ Within the broader field of hospital management, however, Hartwig *et al* described a more specific need for overall operations management in the country’s health centres. Bradley *et al* 5 similarly argue the importance of operations management as a core management competency.^2^ Operations management (sometimes referred to as management science or operations research) can be defined as the application of mathematical techniques to design, understand and improve processes and systems within an organisation.^7^ Some methods, such as simulations and system dynamics, emphasise how changes to parameters or the structure of a system affect its performance; others, such as linear programming or data envelopment analysis, aim to optimise narrower sets of outcomes based on constraints.^7 8^ Compared with traditional management techniques—which often lead to suboptimal decisions—operations management methods can help hospitals improve both their financial health and quality of care.^2–4 9–11^ These methods are also suitable for operational problems and the training of non-technical staff.^12^

Although health systems in high-income countries have adopted these methods, the extent to which they are used in low-income and middle-income countries is unclear. In his bibliography of operations research in Africa, Smith identifies 24 studies in Ethiopia using operations research, with only one which was related to health.^13^ He hypothesised, however, that the apparent scarcity might be due to naming conventions. Practitioners may refer to specific techniques, such as linear programming, rather than operations management research more generally.^13^ In their survey of existing healthcare operations management research, Onar *et al* showed that very few papers on healthcare management came from sub-Saharan Africa.^14^ Health service researchers can often report on operational metrics such as the average length of stay (LOS) or readmission rates in the broader context of a health system, and not in the narrower context of a given hospital. Still, in order to improve and standardise the evidence base, Bradley *et al* call for an expansion of research supporting operations management in resource-constrained settings.^2^ In Ethiopia, the government also supports such a focus, with the Ministry of Health’s Health Sector Transformation Plan emphasising the role of management in healthcare reform.^15^

Despite the limited research in this area, however, there is ample evidence of the need for improved management practices and more efficient hospital operations. Studies at Tikur Anbessa Specialized Hospital (TASH), the largest teaching university hospital in Ethiopia, have reported significant delays in the admission of orthopaedic, oncological and neurosurgical patients.^16–18^ And once admitted, patients can face further delays in treatment that extend their LOS by weeks or months.^16 17^ These delays can put additional strain on existing resources at TASH.

Given this need for operations research, we explored the extent to which operations management methods could be applied to existing routine data sources at TASH. This article presents findings from our experience working with TASH to collect and analyse some of its operational data. We examined two indicators of interest to hospital administrators—LOS and readmissions—in addition to the general problem of forecasting demand for obstetric services. Since our work focused on the utility of existing data rather than an explicit optimisation goal, we eschewed simulation and optimisation techniques in favour of more traditional logistic regressions and time series analysis. We reflected on the challenges of conducting operations research in this setting and conclude with recommendations for researchers and policymakers. Results from this study can guide further operations research work in low-income and middle-income countries, as well as inform future management initiatives.

## Data collection and analysis

We conducted our study in the Department of Obstetrics and Gynecology at TASH in Addis Ababa, Ethiopia. TASH has approximately 600 beds and is the only cancer referral centre in Ethiopia. TASH treats hundreds of thousands of patients in its outpatient departments every year, and it admits thousands more patients for inpatient services. The Department of Obstetrics and Gynecology consists of two outpatient clinics and four inpatient wings. Most inpatient beds are reserved for labour and delivery, but some are maintained for patients with cancer awaiting surgery or chemotherapy.

From August to September 2016, we collected data from paper admission registries of two inpatient wings (table 1): there were 3005 entries for patients admitted between July 2015 and August 2016. Although only 0.3% of entries were missing admission dates, nearly 10% of entries were missing the patient’s medical record number or discharge date. Since the registries are maintained by hand, we first cleaned and de-duplicated the data. After cleaning, the data had 2945 entries.

**Table 1:** Missing data from the ward registries, before and after data cleaning

	Before data cleaning	After data cleaning
Number of observations	3005	2945
Number of missing observations by variable (%)		
Patient MRN	247 (8.2)	247 (8.4)
Age	152 (5.1)	141 (4.8)
City/Region of residence	80 (2.7)	72 (2.4)
Admission date	9 (0.3)	8 (0.3)
Discharge date	281 (9.4)	244 (8.3)
Diagnosis and/or procedure performed	46 (1.5)	34 (1.2)

MRN, medical record number.

Although the registries were sparse, we derived several additional variables. These included the LOS per patient and the number of daily admissions, as well as indicators for the following: living outside Addis Ababa, weekend admission, readmission within 30 days, pre-eclampsia and multiple births. With clean data and a broader feature set, we explored the data’s utility for operational analyses.

## Using existing data to analyse length of stay

There is significant variation in postpartum LOS across low-income and middle-income countries, driven by factors associated with the need for care, such as multiple births and socioeconomic status.^19^ But all hospital stays carry certain risks. Hospital-acquired infections and other adverse events are of concern to hospital administrators. Wilson *et al* 20 determined that 8% of patients in low-income and middle-income countries experienced at least one adverse event; of these events, 30% resulted in death. At TASH, Gedebou *et al* 21 found that 17% of obstetric and gynaecological patients developed an infection. In addition to these infections, vectors such as cockroaches also pose a threat to women in the hospital.^21 22^ By narrowing the range of LOS, administrators may be able to reduce patients’ risk of exposure to adverse events.

We performed two logistic regression analyses with the data (see online supplementary appendix for detailed methodology). First, we examined whether the delivery method was associated with a risk of extended LOS, defined as any LOS more than one SD above the mean LOS. For our second regression analysis, we explored whether the delivery method was associated with a risk of readmission. When patients are readmitted for avoidable reasons, hospitals lose resources that could be better allocated. Patients can lose time, wages or worse. For women giving birth, the delivery method may affect their risk of readmission. Research has found that women who receive a caesarean section face a higher risk of readmission than those with spontaneous vaginal deliveries.^23–25^

10.1136/bmjgh-2018-001281.supp1 Supplementary data

Table 2 shows the characteristics of patients in our study sample who gave birth at TASH. During July 2015–August 2016, 1799 women were admitted at least once to the ward for childbirth: 1529 women (85%) gave birth via caesarean section and 270 (15%) gave birth vaginally. Most vaginal deliveries, however, are discharged from the labour and delivery ward without the need for admission. Our sample, therefore, overestimates the prevalence of caesarean sections, as the women who gave birth vaginally were only admitted due to the presence of complications that warrant an extended stay.

**Table 2:** Descriptive statistics for women giving birth, by birth method

	Caesarean section (n=1529)		Vaginal delivery (n=270)		
	Mean	SD	Mean	SD	t-test
Age (years)	27.7	4.8	27.3	5.0	−1.07
Length of stay (days)	6.3	10.4	4.0	4.8	−3.38***
Daily admissions	8.8	3.1	9.4	3.7	2.65**
	Percentage		Percentage		
Outside Addis Ababa	11.2		16.0		2.21*
Multiple births	1.0		0.4		−0.92
Pre-eclampsia	0.6		1.9		2.15*
Weekend admission	20.0		22.6		0.97
Extended length of stay	10.7		7.0		−2.13*
Readmission after childbirth	1.7		1.1		−0.90

t-tests of differences in means between the two groups were performed. Results were considered significant at the p<0.05 level.

*p< 0.05, **p<0.01, ***p<0.001.

Our sample had a mean LOS of 5.9 days and SD of 9.8 days, so an LOS of greater than 15.7 days was considered to be an extended stay. The mean LOS was 6.3 days among those who had caesarean sections, compared with 4.0 days among women giving birth vaginally (p<0.001). Women delivering via either method had similar mean ages, 28 for caesarean section and 27 for vaginal delivery. Approximately 12% of patients giving birth arrived from outside Addis Ababa, and 20% were admitted during a weekend. Half of all women were discharged in 4 days or fewer, but a quarter remained in the ward for longer than a week.

Table 3 shows our estimated coefficients for the logistic regression model with extended LOS as the outcome. Due to the high level of correlation among some variables and the consequent multicollinearity, 134 observations were dropped from our sample (table 3). Of the covariates included, only age, multiple births and pre-eclampsia had a significant association with the risk of an extended LOS (online supplementary appendix). The model showed no significant difference in risk of extended LOS among patients based on delivery method, daily volume or region of residence. Due to the multicollinearity problem and the small number of readmissions—only 30 women were readmitted after giving birth—our sample was too underpowered to detect any significant patterns related to postpartum readmission (online supplementary appendix table A1).

**Table 3:** Logistic model regression results for the predictors of extended length of stay (n=1665)

Variable	OR	95% CI
Age	1.06**	1.02 to 1.10
Number of admissions per day	1.01	0.95 to 1.07
Outside Addis Ababa	1.14	0.69 to 1.89
Multiple births	5.14*	1.44 to 18.39
Pre-eclampsia	4.47	1.42 to 14.03
Weekend admission	1.08	0.68 to 1.70
Caesarean section	1.52	0.90 to 2.58
Readmission after childbirth	1.19	0.35 to 3.98

P(x>χ^2^)=0.001.**

*p<0.05, **p<0.01.

Still, our results on LOS are directionally consistent with those found by Campbell *et al* 19 in their analysis of national survey data from 92 countries; patients who generally have a greater need for care—such as older patients or patients with multiple births—also have longer postpartum stays. In this regard, although existing data at TASH could not demonstrate causal relationships, they could be useful for internal reporting or as a point of comparison with existing research.

## Using existing data to forecast demand

**Figure 1:**
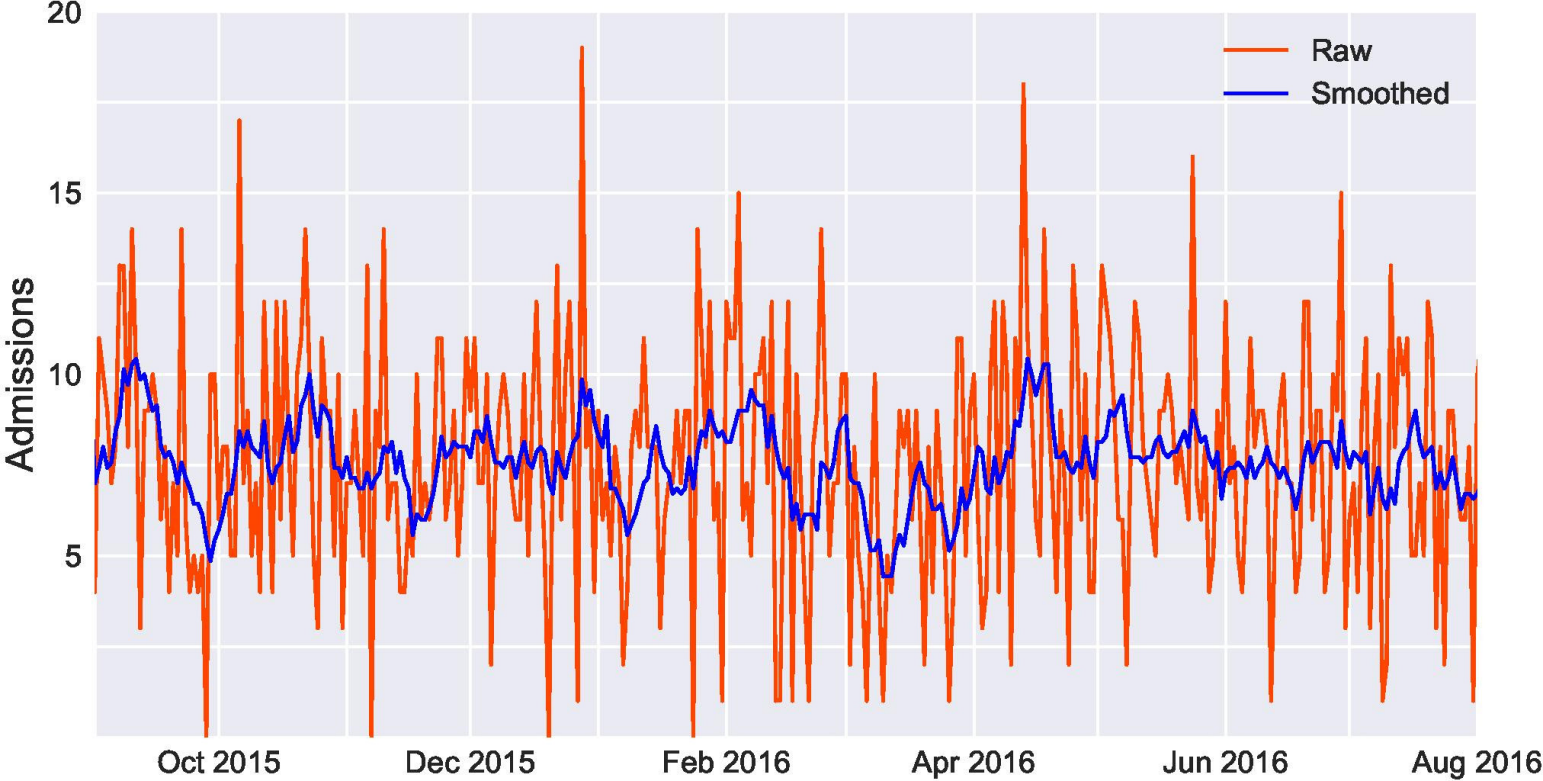
Daily admissions to the obstetrics and gynaecology ward at Tikur Anbessa Specialized Hospital over 2015–2016, showing both raw data and a smoothed 7-day moving average.

**Figure 2:**
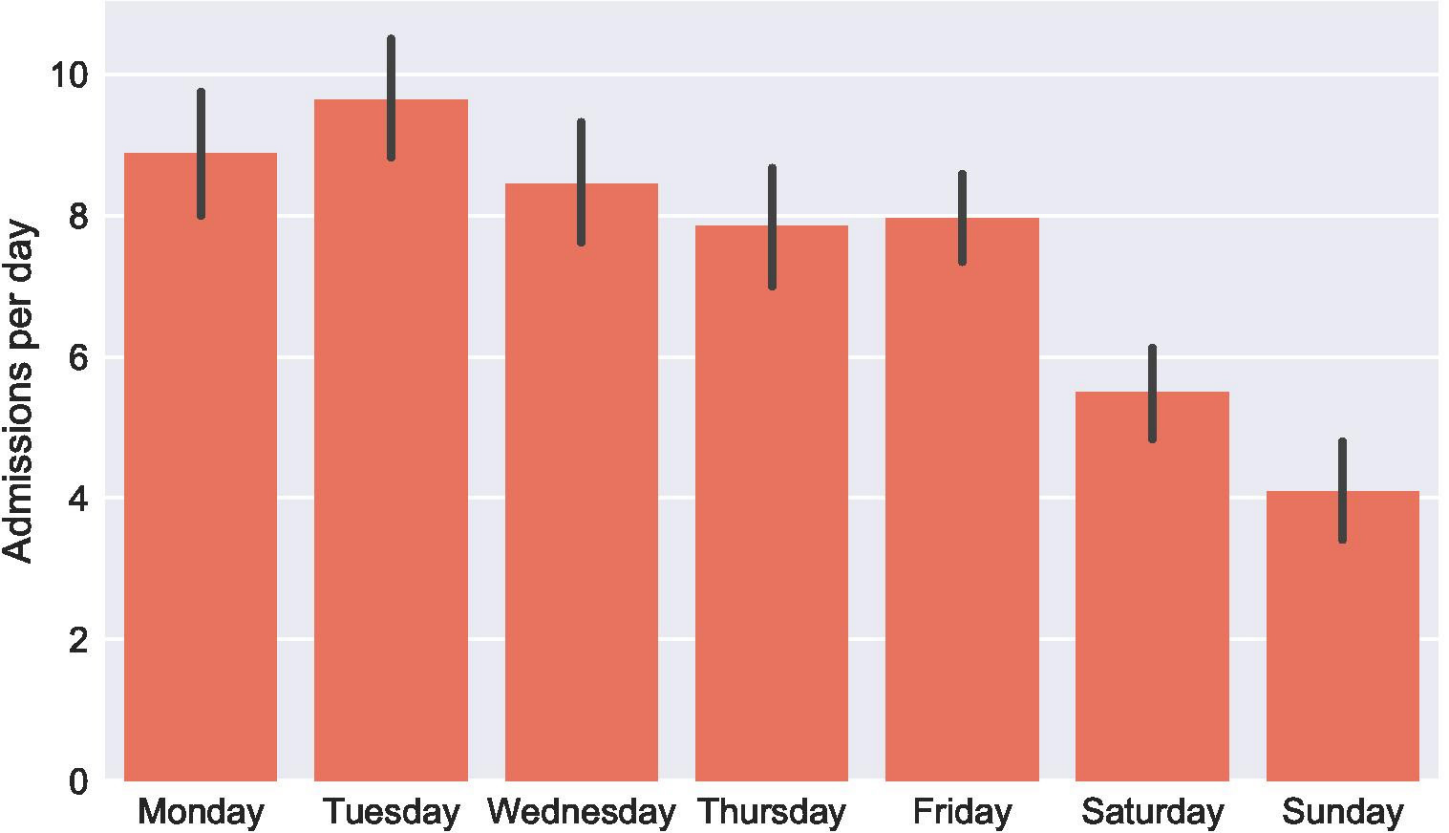
Average number of admissions by day of the week in the obstetrics and gynaecology ward at Tikur Anbessa Specialized Hospital over 2015–2016, with 95% CIs.

We also examined whether basic time series models could effectively predict future demand for services. For facilities trying to match supply to demand, forecasting represents a crucial part of the broader management infrastructure.^8^ Accurate predictions of future demand can help facilities operate more efficiently. Poor forecasts strain staff and resources, leading to time delays, potential errors and suboptimal treatment. The admissions data we used for forecasting spanned approximately 1 year, over July 2015–August 2016 (figure 1). On a given day, the ward would admit anywhere between 15 patients and none at all. Nevertheless, on average, admissions to the ward would follow a predictable pattern by the day of the week (figure 2).

Given the limited data, we restricted our analysis to a few simple models of daily and weekly admissions. For weekly forecasts, the models included a historical mean, a naïve one-step model, and moving averages (MA) and exponentially weighted moving averages (EWMA) with windows of 2, 3 and 4 weeks (see online supplementary appendix table A2 for model details). For daily forecasts, we used the same types of models with a few additions. We included the average admissions by day of the week as well as average admissions on weekdays versus weekends.

For both time scales, we split our data into two parts, a training set and a testing set (see online supplementary appendix for detailed methodology). Three different error metrics were used to evaluate forecast accuracy: mean absolute deviation (MAD), mean forecast error (MFE) and mean absolute scaled error (MASE). MAD describes the accuracy of the forecasts, whereas MFE signals whether the models overestimate or underestimate future demand. MASE is scale-independent, and it can be used to compare daily and weekly forecasts.^26^

Table 4 shows the error metrics for our weekly forecasting models. The constant historical mean performs better than other forecasting methods; all models, however, have MASE values below 1, indicating that they perform better than the naïve one-step model. The historical mean also minimises the MAD. On average, its predictions of weekly volume are off by 6.5 patients per week (compared with 54 weekly admissions in the testing set). Its MFE, however, is approximately three times larger than those for other models. Therefore, the historical mean, like all of the models, tends to underestimate daily demand.

**Table 4:** Metrics for capturing the accuracy of the weekly forecasting models

	Mean	Naïve	MA			EWMA		
			2 weeks	3 weeks	4 weeks	2 weeks	3 weeks	4 weeks
MAD	6.51	8.52	7.89	6.96	7.33	7.73	7.43	7.28
MFE	−1.89	−0.59	−0.63	−0.69	−0.54	−0.61	−0.62	−0.62
MASE	0.76	1.00	0.92	0.82	0.86	0.90	0.87	0.85

Time periods represent window sizes for moving average calculations.

EWMA, exponentially weighted moving average; MA, moving average; MAD, mean absolute deviation; MASE, mean absolute scaled error; MFE, mean forecast error.

Daily forecasting models had similar results (table 5). The ward admitted an average of 7.7 patients per day. The historical mean performed better than the MA and EWMA methods, but it was not the best forecasting method. Both the daily average and the weekday/weekend average models performed better than the historical mean. In general, our daily forecasts had lower MASE values than the weekly forecasting methods. The MAD values also represented significant errors when compared with the average number of daily admissions. Again, the MFE values for all models were negative, indicating consistent underestimation of actual daily volume. Although the three models based on historical averages performed better than the other models, their MFEs reflected a stronger bias towards underestimation than the MA and EWMA models.

**Table 5:** Metrics for capturing the accuracy of the daily forecasting models

				Naïve		MA		EWMA	
	Mean	Daily	Weekend	1 day	7 days	3 days	7 days	3 days	7 days
MAD	2.68	2.57	2.44	3.67	3.26	3.19	2.80	3.15	2.90
MFE	−0.13	−0.40	−0.33	−0.03	−0.07	−0.02	−0.04	−0.03	−0.04
MASE	0.73	0.70	0.67	1.00	0.89	0.87	0.76	0.86	0.79

Time periods represent window sizes for moving average calculations.

EWMA, exponentially weighted moving average; MA, moving average; MAD, mean absolute deviation; MASE, mean absolute scaled error; MFE, mean forecast error.

Using existing data at TASH to inform basic forecasting models could facilitate better staff scheduling and improve bed management. The MFE values in our models, however, illustrate a tendency to underestimate patient volume. In practice, the utility of forecasting models may depend on the cost of underestimating future volume compared with the cost of overestimating future volume. Estimates of patient volume that are too high can result in costs from underutilised staff time and hospital resources; estimates that are too low can result in overburdened staff, and patient care could potentially suffer.^27^ If forecasting models can account for cyclical admission patterns, like the weekend/weekday model, they are likely to be more useful. Other time series techniques such as Fourier analysis may provide additional insight into patterns of patient admissions, but require either more longitudinal or more granular (eg, admissions by hour) data than we were able to collect.

## Challenges and recommendations

In collecting and analysing our data, we faced several obstacles which apply more broadly to operational research in low-income and middle-income countries. First, most records at TASH were kept in paper files. Research with paper records, whether it involves patient chart reviews or data entry from admissions registries, is very resource-intensive. Additionally, these systems are not robust against errors. Paper records can suffer from environmental degradation (eg, torn pages) as well as human error (eg, incorrect transcription).

Second, as shown in table 1, even fairly standard administrative records were missing significant amounts of data. The quality of the data that were recorded could be questioned. For example, data on a patient’s stay were sometimes duplicated among registries; cleaning and de-duplicating the data took a significant amount of time and resources. Paper records also restrict the number of variables that analysts can mine for operational insights. In light of these challenges, we propose the following recommendations.

### Improve existing information systems

Our analysis illustrates the limitations of paper-based records. Still, strategies to improve paper-based record systems have been developed by other researchers.^28^ In their review of paper registries in four countries, including Ethiopia, Westley *et al* 28 recommend that only essential data be collected and that registries be designed to fit local needs. Additionally, internal systems could audit data quality and incorporate feedback from front-line users into registry design, for example.^28^ Efforts to improve information systems in Mozambique and Rwanda have pursued both of these strategies.^29^ By critically examining data collection processes, managers can better understand how they contribute to broader organisational goals.

### Expand educational and training programmes

Hospitals in Ethiopia have already benefited from the adoption of operations management techniques.^2–4^ Much of the training, however, has only targeted executives.^3 4 30^ For operations management to become standard practice, this training should be extended further to hospital administrative staff. Ethiopia has made progress in expanding access to educational opportunities in this area. Prior to 2009, no graduate programme in healthcare administration was offered in the country.^31 32^ By 2016, there were five masters in healthcare administration programmes across the country.^31^ These programmes—which combine coursework with fieldwork—will be crucial to the dissemination and implementation of operations management methodologies.

### Integrate operations management into broader hospital management

Compared with operations management methodology, traditional management techniques often lead to suboptimal decisions.^11^ By adopting operations management techniques, hospitals can improve both their financial health and quality of care.^2–4 9 10^ For such changes to take root, however, physicians and hospital leaders must adopt these techniques and integrate them into day-to-day operations. This could be achieved in a variety of ways, such as the establishment of an analytics group within the hospital. In our collaboration with staff at TASH, we received feedback from clinicians that operationally focused analytics could help the ward allocate resources more effectively and efficiently, enabling better patient care. This type of stakeholder engagement and buy-in is necessary for implementing operations management initiatives.

## Conclusion

Integrating operational data and operations management methods into hospital management can improve the efficiency of healthcare delivery in low-income and middle-income countries. However, as we found in this analysis, many obstacles must first be overcome. Although extant data sources can provide basic insights, the collection and maintenance of high-quality data by hospital staff must also be prioritised in order for operations management methods to yield their greatest returns.
