# Supplementary material for: Modelling hospital operations: insight from using data from paper registries in the obstetrics ward at a hospital in Addis Ababa, Ethiopia
Source: BMJ Glob Health. 2019 May 16;4(3):e001281. doi: 10.1136/bmjgh-2018-001281 (PMC6528765; doi:10.1136/bmjgh-2018-001281)
Supplement: Supplementary data [file bmjgh-2018-001281supp001.pdf]

Supplementary appendix

Logistic regression models

We performed two logistic regression analyses with the data. Table 3 shows our estimated coefficients for the logistic regression model with extended LOS as the outcome. Due to the high level of correlation among some variables and the consequent multicollinearity, 134 observations were dropped from our sample. Our model had a *p*-value for Chi-squared goodness-of-fit statistic of less than 0.001. Of the covariates included, only age, multiple births, and pre-eclampsia had a significant association with the risk of an extended LOS. Older patients seem to remain in the ward longer than younger ones; a one-year increase in age was associated with a 6% (95% CI: 1.02-1.10) increase in the risk of an extended LOS. Patients giving birth to more than one child were over 5 (1.44-18.39) times more likely to have an extended LOS compared to patients giving birth to one child. Similarly, patients with pre-eclampsia had an odds ratio of 4.47 (1.42-14.03) for an extended LOS. The model showed no significant difference in risk of extended LOS among patients based on delivery method, daily volume, or region of residence. We found no significant results in our analysis of the risk of readmission (Table A1).

Our analysis showed that patients with multiple births at TASH had an OR for extended stays of 5.14. Campbell et al. found that having twins or triplets was associated with an average increase in LOS of close to two days.[20] Although the effect of urban/rural residence matches analyses from Campbell et al., our finding of a null effect of delivery method on postpartum LOS may be an artifact of the sample, as their analysis found that patients giving birth via Caesarean section had longer LOS after adjusting for other factors.[20]

Table A1. Logistic regression results, examining predictors of readmission after childbirth (N =1,449).

Variable	Odds ratio	95% confidence interval
Age	1.04	(0.98, 1.10)
Number of admissions per day	0.99	(0.86, 1.14)
Outside Addis Ababa	1.00	Dropped due to collinearity
Multiple births	1.00	Dropped due to collinearity
Pre-eclampsia	1.00	Dropped due to collinearity
Weekend admission	1.01	(0.39, 2.66)
Caesarean section	2.22	(0.52, 9.44)
Extended LOS	1.17	(0.35, 3.94)

Note. $P(x>\chi^2) = 0.610$. LOS = length of stay. Three variables were dropped from the model due to a high level of correlation among them and the outcome variable.

Time series forecasting models

The training set for daily forecasts consisted of admissions in August 2015. Admissions between September 2015 and August 2016 formed the testing set. At the weekly level, the training set spanned from August 2015 to January 2016, a period of six months. The subsequent six-month period, from February to August 2016, formed the testing set. See Table A2 for details on model formulations.

Table A2. Mathematical formulations of the time series forecasting models.

Model	Description	Formula
Historical mean	The historical mean is the average of all admissions over a given time period *T*. The time period used to calculate the historical mean for daily admissions was August 1, 2015 to August 31, 2015. The time period used to calculate the historical mean for weekly admissions was August 2015 to January 2016.	$\hat{Y}_{t+1} = \frac{\sum_{i=1}^{T} Y_i}{T}$

Naïve forecast	The naïve, or one-step, forecast predicts the value $\hat{Y}$ at time $t + n$ to be the actual value of Y in the previous period t. For weekly forecasts, $n = 1$. For daily forecasts, $n = 1$ and 7, i.e. steps of one day and one week.	$\hat{Y}_{t+n} = Y_t$
Moving average (MA)	The moving average predicts $\hat{Y}$ in the period $t + 1$ to be the average of actual values during the previous N periods, where N is called the window size. For the weekly forecasts, we tested window sizes of 2, 3, and 4 weeks. For daily forecasts, we tested window sizes of 3, 5, and 7 days.	$\hat{Y}_{t+1} = \frac{\sum_{i=t-N}^{T} Y_i}{N}$
Exponentially weighted moving average (EWMA)	The exponentially weighted moving average (EWMA) discounts past values of Y and $\hat{Y}$ according to a scaling factor α. The scaling factor α can be written as a function of a window with N periods. For daily forecasts, we tested window sizes of 3, 5, and 7 days. For the weekly forecasts, we tested window sizes of 2, 3, and 4 weeks.	$\hat{Y}_{t+1} = \alpha Y_t + (1-\alpha)\hat{Y}_t$<br>$\hat{Y}_0 = Y_0$<br>$\alpha = \frac{2}{1+N}$
Daily historical mean	The daily historical mean model calculates mean admissions $\bar{Y}$ by day D of the week in the training period and applies those means to the testing period. The predicted number of admissions on a Wednesday, for example, is the average number of admissions on Wednesdays in the training set. The training period for daily admissions was August 2015. The training period for weekly admissions was August 2015 to January 2016.	$\hat{Y}_{t+1} = \bar{Y}_D$
Historical mean for weekdays and weekends	The model predicts $\hat{Y}$ on day D as the mean daily admissions $\bar{Y}_W$ during either weekdays or weekends, depending on D, in the training period. We considered Monday through Friday to be weekdays and Saturday and Sunday to be weekends. The training period for daily admissions was August 2015. The training period for weekly admissions was August 2015 to January 2016.	$\hat{Y}_{t+1} = \bar{Y}_W$
